# High performance of the automated ADVIA Centaur Systems SARS-CoV-2 Antigen Assay in nasopharyngeal samples with high viral load

**DOI:** 10.1007/s10123-022-00311-3

**Published:** 2022-12-17

**Authors:** Esther Ríos, Sara Medrano, Mar Alvarez, María José Valderrama, Luis Vallejo, Alberto Delgado-Iribarren, Esther Culebras

**Affiliations:** 1 Department of Medicine, Facultad de Medicina, Universidad Complutense Madrid, Plaza Ramón y Cajal S/N, Madrid, 28040 Spain; 2 Department of Clinical Microbiology, Instituto de Investigación Sanitaria Hospital Clínico San Carlos, IdISSC, Madrid, Spain; 3 Department of Genetics, Physiology and Microbiology, Faculty of Biology, Universidad Complutense de Madrid, Madrid, Spain

**Keywords:** SARS-CoV-2, Rapid antigen test, PCR, Sensitivity, Specificity

## Abstract

ADVIA Centaur SARS-CoV-2 Antigen (COV2Ag) Assay (Siemens Healthineers) was evaluated for SARS-CoV-2 detection. A total of 141 nasopharyngeal samples were analyzed by this technique and results were compared with those obtained by quantitative reverse-transcription polymerase chain reaction (RT-PCR). The overall sensitivity and specificity of the test were 68.70% and 70%, respectively. Regarding cycle threshold (Ct) values, the COV2Ag test showed a sensitivity of 93.75% and 100% for nasopharyngeal samples with Ct < 25 and < 20, respectively. ADVIA Centaur COV2Ag Assay is a useful, automated, and rapid technique for early SARS-CoV-2 diagnosis and isolation of the infected individuals, avoiding its transmission.

## Introduction

The “Severe Acute Respiratory Syndrome Coronavirus 2 (SARS-CoV-2)” disease has caused a threatening pandemic globally (COVID-19) (Meo et. al. 2021). The rapid diagnosis of COVID-19 patients is essential to reduce the disease spread. To date, the reverse-transcription polymerase chain reaction (RT-PCR) is the gold standard technique in routine clinical practice to diagnose SARS-CoV-2 infection (Kevadiya et al. 2021). RT-PCR is a high-cost technique and requires specialized equipment and professionals. Therefore, it is necessary to provide automated and rapid detection methods.

The SARS-CoV-2 Antigen (COV2Ag) tests are faster and easier to perform and provide a screening to identify infections and control the transmission of the virus (Peck Palmer et al. 2022). In fact, the WHO has recommended their use to investigate certain outbreak situations, to monitor disease trends in communities, and for early detection and isolation of infected individuals in setting where there is a high degree of community transmission (WHO 2020).

The aim of this study was to assess an automated COV2Ag test, ADVIA Centaur COV2Ag Assay (Siemens Healthineers), and compare the results with those obtained by quantitative RT-PCR.

## Materials and methods

A total of 141 samples were analyzed at the Microbiology Department of Hospital Clínico San Carlos in the form of nasopharyngeal swabs in several transport mediums, mainly universal transport medium (UTM), viral transport medium (VTM), and liquid Amies medium. The samples were recovered from June 2020 to June 2021.

The ADVIA Centaur COV2Ag Assay is an automated sandwich immunoassay that uses mouse monoclonal antibodies to detect SARS-CoV-2 nucleocapsid antigen. The system reports results as index values and results are considered negative (index value < 1.0) or positive (index value ≥ 1.0) according to the manufacturer. Calibration and quality control materials were within manufacturer’s specifications. There is a direct relationship between the amount of SARS-CoV-2Ag and the number of relative light units (RLU) (TCID50/mL) detected by the system.

In order to detect SARS-CoV-2 by RT-PCR, RNA was extracted by NucliSENS easyMAG™ method (bioMérieux, Madrid, Spain). PCR amplifications were performed using the TaqPath™ Multiplex RT-PCR COVID-19 kit in a QuantStudio5 thermocycler (Thermo Fisher Scientific) according to the manufacturer’s protocol. Samples were considered positive with cycle threshold (Ct) values < 40.

Statistical analyses were performed using GraphPad Prism v.5.01 to compare index values of the COV2Ag and RT-PCR results. Student’s *t* and Bonferroni’s multiple comparison tests were used to assess differences of CoVAg index values among the RT-PCR positive and negative samples and between RT-PCR Ct values, respectively. A *P*-value < 0.05 was considered statistically significant.

## Results

The results of the COV2Ag Antigen Assay and RT-PCR are shown in Table 1. Using RT-PCR as the reference method, the sensitivity and specificity of the COV2Ag test for the diagnosis of COVID-19 in nasopharyngeal swabs were 68.70% and 70%, respectively. Only three samples were considered false-positive by the COV2Ag Assay with an index range from 1.41 to 5.83.

**Table 1:** Comparison of the results between the SARS-CoV-2 antigen test and RT-PCR

		RT-PCR		
		Positive	Negative	Total
COV2Ag	Positive	90	3	93
	Negative	41	7	48
	Total	131	10	141

Sensitivity: 68.70%; Specificity: 70%

Regarding the viral load (Ct values) determined by RT-PCR, the sensitivity of the COV2Ag Assay increased to 93.75% for samples with Ct values < 25 (Table 2). In fact, for RT-PCR positive samples with Ct values < 20, the COV2Ag exhibited 100% sensitivity.

**Table 2:** Sensitivity according to the Ct value of RT-PCR

RT-PCR Ct value	RT-PCR positive results	COV2Ag positive results	Sensitivity (%)
< 20	28	28	100
< 25	64	60	93.75
< 30	101	88	87.13
All (< 40)	131	90	68.7

**Fig. 1:**
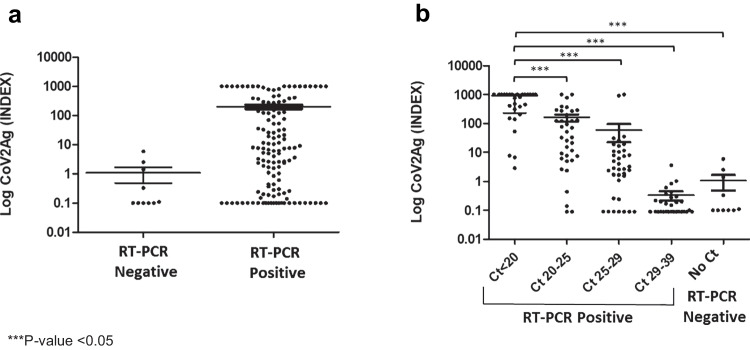
SARS-CoV-2 antigen detection according to RT-PCR results. **a** Distribution of CoVAg index values according to RT-PCR results. **b** Distribution of CoVAg index values according to Ct values. Data are presented as mean ± SEM

The mean SARS-CoV-2 Ag index value among the RT-PCR positive samples was significantly higher (200.0 ± 30.78) than that of the RT-PCR negative samples (1.067 ± 0.5865) (Fig. 1a). Results were statistically significant (*P*-value = 0.0386), indicating that the antigen test was able to distinguish between positive and negative samples for SARS-CoV-2.

Interestingly, comparing index results of the COV2Ag Assay between Ct values (Fig. 1b), statistical analysis revealed significant differences between samples with Ct < 20 and those samples with a Ct > 20 (even RT-PCR negative samples). Conversely, there was no significant difference between the rest of the groups of Ct values regarding the SARS-CoV-2 Ag index values.

## Discussion

In the present study, we evaluated an automated and rapid test for the diagnosis of COVID-19 in nasopharyngeal swabs. Using RT-PCR as the reference method, the sensitivity and specificity of the ADVIA Centaur Systems SARS-CoV-2 Antigen Assay were 68.70% and 70%, respectively. Several antigen tests have previously been evaluated for a first screening in the diagnosis of SARS-CoV2, and revealed a sensitivity and specificity ranging from 37 to 90% and 65 to 100%, respectively (Khalid et al. 2022). In the case of ADVIA Centaur COV2Ag Assay, two previous investigations demonstrated a higher overall sensitivity (88.5% and 82.4%) and specificity (95.5% and 97.3%) than those found in our study (Hörber et al. 2022; Peck Palmer et al. 2022).

Antigen test agreement with RT-PCR has been described to be higher at lower Ct values (Altawalah et al. 2021; Hirotsu et al. 2020; Hörber et al. 2022; Jeewandara et al. 2022; Khalid et al. 2022; Peck Palmer et al. 2022). Therefore, we also considered evaluating the COV2Ag test according to the Ct values. For RT-PCR positive samples with Ct values < 25, the sensitivity of the COV2Ag Assay increased to 93.75%. Ct values < 25 have been associated with higher viral loads and higher infectivity; hence, they are important tools in identifying infectious individuals in the community (Jeewandara et al. 2022). Regarding samples with Ct values < 20, the COV2Ag exhibited 100% sensitivity. These results are in line with the manufacturer’s claims and a previous study by Hörber et al. (2022) that demonstrated a good performance of the Siemens Healthineers COV2Ag Assay in samples with high viral load (Ct value < 20).

One of the limitations of this study is that the performance of this assay was not evaluated according to the symptom status and duration. As described (Peck Palmer et al. 2022), concordance between COV2Ag and RT-PCR could vary between symptomatic and asymptomatic patients. Nevertheless, these data were not recorded in this study.

In conclusion, the COV2Ag Assay showed a high performance for SARS-CoV-2 diagnosis in samples with high viral loads (Ct < 25), even displaying 100% sensitivity in samples with Ct < 20. Furthermore, ADVIA Centaur COV2Ag Assay is an automated, easy to use, and fast technique with a time to first result in as little as 26 min. Therefore, it is useful for early SARS-CoV-2 diagnosis and isolation of the infected individuals, avoiding its transmission.
